# Communication between mothers and health workers is important for quality of newborn care: a qualitative study in neonatal units in district hospitals in South Africa

**DOI:** 10.1186/s12887-019-1874-z

**Published:** 2019-12-16

**Authors:** Christiane Horwood, Lyn Haskins, Silondile Luthuli, Neil McKerrow

**Affiliations:** 1 Centre for Rural Health, University of KwaZulu-Natal, George Campbell Building, Howard College Campus, Durban, South Africa; 2 KwaZulu-Natal Department of Health, Durban, South Africa; 3 Department of Paediatrics and Child Health, University of KwaZulu-Natal, Durban, South Africa

**Keywords:** Quality care, Neonatal care, Maternal care, Patient-provider communication, Person centred care, Africa, South Africa, Newborn care, Infant mortality, Effective communication

## Abstract

**Background:**

There is a high global burden of neonatal mortality, with many newborn babies dying of preventable and treatable conditions, particularly in low and middle-income countries. Improving quality of newborn care could save the lives of many thousands of babies. Quality of care (QoC) is a complex and multifaceted construct that is difficult to measure, but patients’ experiences of care are an important component in any measurement of QoC. We report the findings of a qualitative study exploring observations and experiences of health workers (HWs) and mothers of babies in neonatal units in South Africa.

**Methods:**

A qualitative case study approach was adopted to explore care of newborn babies admitted to neonatal units in district hospitals. Observation data were collected by a registered nurse during working hours over a continuous five-day period. Doctors and nurses working in the neonatal unit and mothers of babies admitted during the observation period were interviewed using a semi-structured interview guide. All interviews were audio recorded. Observation data were transcribed from hand written notes. Audiotapes of interviews were transcribed verbatim and, where necessary, translated into English. A thematic content analysis was used to analyse the data.

**Results:**

Observations and interviews were conducted in seven participating hospitals between November 2015 and May 2016. Our findings highlight the importance of information sharing between HWs and mothers of babies, contrasting the positive communication reported by many mothers which led to them feeling empowered and participating actively in the care of their babies, with incidents of poor communication. Poor communication, rudeness and disrespectful behaviour of HWs was frequently described by mothers, and led to mothers feeling anxious, unwilling to ask questions and excluded from their baby’s care. In some cases poor communication and misunderstandings led to serious mismanagement of babies with HWs delaying or withholding care, or to mothers putting their babies at risk by not following instructions.

**Conclusion:**

Good communication between mothers and HWs is critical for building mothers’ confidence, promoting bonding and participation of mothers in the care of their baby and may have long term benefits for the health and well-being of the mother and her baby.

## Background

There is a high burden of neonatal mortality globally, with an estimated 7000 infants dying every day. Despite considerable progress in improving child mortality in recent years, similar reductions have not been achieved in reducing neonatal mortality, so that newborn deaths now account for 45.1% of all deaths in children under five years [1]. In many countries, including South Africa, many infants die from preventable causes despite the availability of proven, cost-effective interventions to manage the major causes of newborn deaths [2, 3]. In addition, poor neonatal care may lead to substantial morbidity and long-term disability [4]. It is estimated that improved quality of newborn care could save the lives of thousands of infants in South Africa [5], particularly in district hospitals where many infant deaths occur [6].

Strengthening health systems and improving adherence to evidence-based guidelines can improve quality of clinical care, and may lead to improved patient outcomes [7, 8]. However, quality of care (QoC) is a complex construct that is difficult to define and measure, and goes beyond evidence-based clinical care and adherence to treatment protocols. Other dimensions of quality may include care that is safe, efficient, patient-centred, equitable and cost-effective, depending on the perspective of the assessor [9, 10]. This complexity makes it difficult to determine how best to measure such a crucial and multi-facetted concept [11].

Many studies exploring QoC have used quantitative methodologies, such as inventories, checklists clinical audit and facility reviews to determine availability of resources including staff, equipment, consumables and drugs [12–14]. Although these elements form the foundation without which good quality care cannot be provided, evidence suggests that other key components of quality, particularly those related to interpersonal dimensions of care, such as communication, shared decision-making and patient satisfaction, are also important and can influence outcomes [15–17]. Adding the perceptions of HWs and patients to an assessment of QoC allows for a deeper understanding of underlying factors, which may explain why good practices are not followed, identify avoidable factors and provide direction for improving quality of care [11, 18]. These components of quality may be difficult to measure and less valued than clinical competency [19], and have frequently been neglected in studies exploring and monitoring QoC [11, 12]. However, it is being increasingly recognized that any framework for QoC should include interpersonal aspects [18, 20, 21], and that experiences and perceptions of quality of care are key drivers of service utilization [22, 23].

Care of sick newborn infants in the neonatal unit requires complex equipment and technical skills, with the result that assessments of QoC in the neonatal unit may focus strongly on provision of medical treatment according to clinical guidelines, rather than on the interpersonal aspects of care. However, the intensive, stressful and technical environment in the neonatal unit can be a barrier to effective communication between health workers (HWs) and mothers, and to a mother’s involvement in the care of her baby [15]. Studies have shown that the admission of her baby to the neonatal unit can lead to severe maternal distress, with mothers describing the experience as devastating, traumatic and life-altering [24]. Thus, effective communication and collaborative engagement between health professionals and mothers should be considered an essential component of providing good quality care for the mother and her baby.

However, poor patient-provider interactions, lack of information, disrespectful and abusive care have been widely reported, including in African settings, and are recognized as an important barrier to accessing care, particularly maternity care [16, 19, 23]. Lack of involvement in the baby’s care may lead to a loss of parental role and disrupt bonding between the mother and baby at a critical time, which may affect the mothers physical and mental health [25], and have a negative impact on the baby’s long-term physical and cognitive development [26]. In order to identify such behaviours and address these concerns, a framework has been developed defining elements of disrespect and abuse during childbirth, including neglect and abandonment, non-consented care, non-confidential care, non-dignified care as well as physical abuse [16]. The respectful maternity care charter sets out the rights of childbearing women including the right to information, informed consent, privacy and confidentiality, and respectful treatment [21]. The World Health Organization (WHO) acknowledges the right of women to dignified and respectful care [21], and it has been argued that the charter should include both parts of the mother-infant dyad, and therefore, that the right to respectful care should be extended to the newborn [27].

A comprehensive assessment of quality of newborn care should include developing an in-depth understanding of how care is provided, and gaining different perspectives on factors contributing to poor quality of care [22]. This requires a holistic methodology with qualitative research techniques employed to explore multiple dimensions of care provision. Combining observations and interviews provides deeper insights and is a useful method to identify ways to improve quality within existing resources [11]. This study forms a component of a multipronged initiative to improve QoC in neonatal units in district hospitals in KwaZulu-Natal (KZN), known as the KZN Initiative for Newborn Care (KINC) [28]. The KINC intervention is described in detail elsewhere [29, 30]. We report the findings of an in-depth qualitative study which aimed to explore mothers and HWs experiences of care provided in neonatal units in district hospitals in order to identify opportunities to improve QoC.

## Methods

An observational, qualitative case study approach was adopted to explore care provision for small and sick newborn babies admitted in district hospitals in KZN. A case study design is a qualitative approach that is flexible enough to capture the complexity of the study setting by using multiple data collection methods to triangulate findings thereby providing in-depth understanding of the case. We used neonatal units as cases to provide in-depth insight into experiences of care using a case study methodology [31]. The study was conducted at the end of the KINC initiative after completion of all KINC activities.

### Study setting

The study was conducted in selected district hospitals in KZN province, South Africa. KZN is one of 11 provinces in South Africa and has a population of approximately 11 million people [32]. In KZN the proportion of deliveries in a health facility is 94.8 and 34.0% of neonatal deaths occur in district hospitals (SA National Department of Health Information System [DHIS] data, personal communication Dr. Neil McKerrow). At the time of the study, there were 39 district hospitals, 10 regional hospitals and one tertiary hospital in KZN providing care to newborn babies. The early neonatal mortality rate was estimated at 10.6 per 1000 live births from DHIS data in 2015 [33]. This paper focusses on district hospitals, which provide generalist health services and support to primary health care clinics within a sub-district. By definition, medical care for babies in district hospitals is provided by generalist medical practitioners, supported by regular outreach visits from paediatricians from regional or tertiary hospitals. Care in the neonatal unit is provided by a team of nurses under the guidance of an advanced midwife, a nurse with specialist midwifery training that includes neonatal care. High care services, including continuous positive airways pressure (CPAP) but not intensive care or artificial ventilation, are provided at district hospitals. Intensive care is provided at regional referral hospitals, often located several hours away from the district hospitals.

Health workers strongly encourage mothers of babies in the neonatal unit to feed the baby breastmilk, either breastfeeding or by giving expressed breastmilk via nasogastric tube. Mothers usually stay in the hospital for the duration of the baby’s admission, and are requested to come to the neonatal unit at regular intervals during the day to feed the baby. Mothers also provide general care for the baby, changing nappies and washing the baby if appropriate, and have to comply with unit policies.

### Sampling

#### Selection of district hospitals

The sampling frame was all 39 district hospitals providing neonatal care in KZN. Cases were individual neonatal units and these were purposively selected to provide a range of experiences of quality of care in differently performing hospitals. Firstly, we excluded very small hospitals with < 1500 deliveries annually, because neonatal units in these hospitals frequently have very few or no babies admitted. The second step was based on our previous work where quantitative surveys were used to measure quality of newborn care in district hospitals over time [29]. We selected the two highest scoring hospitals; two lowest scoring hospitals; two hospitals with the most improved score and two hospitals with the least improved (or reduced) score. One hospital fell into two categories, so seven hospitals were included in the study sample (Table 1).

**Table 1:** Selection of participating hospitals

	Deliveries per annum	Number of neonatal beds	Rural/Urban	KINC Score at midpoint/88	Reason for selection
Hospital 1	3750	11	Urban	72.0	Overall highest QoC score at midpoint
Hospital 2	3500	11	Rural	66.4	Second highest QoC score at midpoint
Hospital 3	2750	8	Rural	61.6	Biggest improvement in QoC score from baseline to midpoint
Hospital 4	3000	9	Rural	60.3	Second biggest improvement in QoC score from baseline to midpoint
Hospital 5	3500	11	Rural	53.5	Least improvement in QoC score from baseline to midpoint
Hospital 6	5500	17	Urban	42.3	Second least QoC score at midpoint AND lowest improvement in QoC score from baseline to midpoint
Hospital 7	2500	11	Rural	39.2	Overall lowest QoC score at midpoint

#### Selection of mothers and HWs

All mothers with babies admitted to the neonatal unit during the observation period, and all nurses and doctors working in the nursery during the observation period were requested to participate in the study. Mothers were excluded if they were under 18 years or if they were unable to speak a local language (English or IsiZulu).

### Data collection

A trained field worker, who was a registered nurse and midwife, conducted structured observations in the neonatal unit during working hours over a continuous five-day period (Monday to Friday). A ‘15-minute’ tool was used as an aid to writing field notes so that verbal and non-verbal interactions were documented as they occurred in the neonatal unit over each 15-minute period on each day of observation. Collecting data continuously over five consecutive days made observational data more reliable, and observation techniques provide a gold standard for clinical quality assessment and understanding behaviours [34].

HWs working in the neonatal unit (doctors and nurses) and mothers of babies admitted in the neonatal unit during the period of observation were interviewed by experienced qualitative researchers on day 3–4 of the five-day observation period, using a semi-structured interview guide. All interviews were audio recorded. Mothers were predominantly isiZulu speaking and were interviewed in isiZulu or English, whichever they preferred. All HW interviews were conducted in English.

### Ethics

Ethical approval was obtained from the University of KwaZulu-Natal, Biomedical Research Ethics Committee (BE 177/13) and from the KZN Department of Health (DoH). Permission to undertake the study was granted by the KZN DoH and the Chief Executive Officers (CEOs) of each hospital. All participating mothers and HWs provided written informed consent. To maintain confidentiality and anonymity participants were identified by a code and no personal identifiers were collected. Data were stored in a password protected file.

### Data analysis

Observation data were transcribed from hand written notes collected using the 15-minute tool. Audiotapes of interviews were transcribed verbatim and, where necessary, translated into English. A thematic content analysis was used to analyse the data, observation data and interview data were analysed together and triangulation was used to validate the data from each source. Data analysis proceeded iteratively with two researchers working together to identify the main themes emerging from the data. To ensure trustworthiness, two researchers identified and discussed their different perceptions and reached an agreement on the data interpretations and theme descriptions. As analysis proceeded emerging themes were identified and added as sub themes to the main themes. Data was coded using computerized qualitative data analysis software (NVivo version 10.0).

## Results

In each of seven participating hospitals, observations were conducted during working hours (8 am-4 pm) over a continuous five-day observation period, between November 2015 and May 2016. In addition, 24 interviews were conducted with mothers of sick babies and 20 interviews with HWs working in the neonatal unit (Table 2). The results presented combine the findings from the thematic analysis of observations and interviews conducted in participating hospitals. Mothers’ described important topics for information sharing in relation to three sub-themes: Results describe mothers’ perceptions of quality of care based on her observations of health workers behaviour and the care provided to her baby, and her experiences of communication and information sharing with HWs. We then contrast this with perceptions expressed by HWs of quality of care and behaviour of mothers. We were unable to find any association between the quantitative performance of participating hospitals and quality of care perceived by participants. Quotes are linked to the hospital numbers shown in Table 1.

**Table 2:** Demographic characteristics of participating mothers and health workers

MOTHERS	*n* = 24
Age Group	
15–20 years	5
21–25 years	8
26–30 years	8
31–35 years	3
Race	
African	23
Indian	1
Baby Information	
Pre-Term	10
Post-Term	2
Full-Term	12
HEALTH WORKERS	*N* = 20
Age Group	
Younger than 30	3
30–40 years	5
40–50 years	10
50–60 years	2
Race	
African	15
Indian	2
White	1
Cadre of HW	
Doctor	7
Professional Nurse	12
Unspecified	1
KINC Training	
Yes	9
No	11

### Mothers’ perceptions of care

#### Observed care

Mothers’ reports indicated that they often based their perceptions of the quality of care received by their babies on the activities of the HWs that they observed while in the neonatal unit. Most mothers expressed that they felt secure in the neonatal unit environment, and were satisfied with the care provided, particularly when they could see the improvements in their baby’s condition. ‘*I love it because it is always warm and I can see that my baby is growing’ (mother, Hospital 5).* Mothers felt that the nurses cared for their baby when they observed them taking care of the baby’s physical needs, taking temperatures, giving medication, changing the linen. They also liked to see the HWs overseeing and monitoring the babies, and were satisfied with the quality of care when they saw monitors and machines connected to their babies.

***MOTHER****: do you see those machines that they use? You sometimes see that they are all plugged next to your baby’s bed, then you see them removed because your baby is better, that is what I have seen and I like. And there are always staff there, busy treating our babies (Hospital 7).*

When mothers observed HWs soothing their babies when they were upset, rather than leaving them to cry, or calling the mothers when the baby needed the nappy changed, this made the mothers feel that babies in the neonatal unit were cared for.

***MOTHER:*** *I can say that they take good care of the baby because sometimes when I go there to breastfeed I find that the baby was crying and the sister has him, quieting him. I can say I really like that (Hospital 4).*

In contrast a few mothers expressed dissatisfaction with the quality of care when they observed health workers failing to provide care timeously or leaving the baby to cry.

***MOTHER:*** *(what I don’t like) is that if we are not present and the baby is crying, he just cries until he stops especially when it’s not the time for us to go in the nursery (Hospital 7).*

#### Communication

Another important factor that determined mothers experiences and perceptions of the care their babies received was the quality of information sharing and dialogue between themselves and HWs. Mothers frequently commented that HWs varied in the way they communicated, they described some HWs as ‘friendly’ and easy to talk to (Case Study 1). Mothers felt free to ask questions about the health of the baby from these HWs, whereas other HWs were rude, appeared to withhold information and, in some cases, were unwilling to answer the mother’s questions. In some cases this poor communication put the baby at risk as described by this mother:

***MOTHER:*** *As you know they take shifts, so you find that some (nurses) talk nicely and some don’t talk. She (nurse) would keep quiet until she goes (home). She doesn’t say a thing, she doesn’t tell you if, maybe, the baby’s amount of feed intake has increased. When the right one arrives, she asks you if you saw that the doctor wrote that the weight has increased and how much is it? I would say “no I did not know I’m still giving him the same amount of feed”, then I increased it after I’ve been told by the nurse who was off for 7 days (Hospital 4).*

Mothers described important topics for information sharing in relation to the clinical condition and progress of the baby, the mother’s own role in caring for her baby on the neonatal unit, and information about tests or procedures to be undertaken on the baby.

##### Communication about the clinical condition of the baby

Most mothers described positive experiences related to communication between themselves and HWs, and mothers described feeling satisfied that care was good when they were given information about the health and progress of their baby, when nurses listened to their concerns, and communicated with them in a friendly and respectful manner.

***MOTHER:*** *What I like the most is that the nurses are always close to the babies, they never lose sight of them and they always tell you should there be any changes on the baby. Even when they were inserting drips they explained to us that drips do cause swollen skin but should it happen that it gets swollen, they will be there to take it out. They even explain to us how the drip functions (Hospital 2)*

**Fig. 1:**
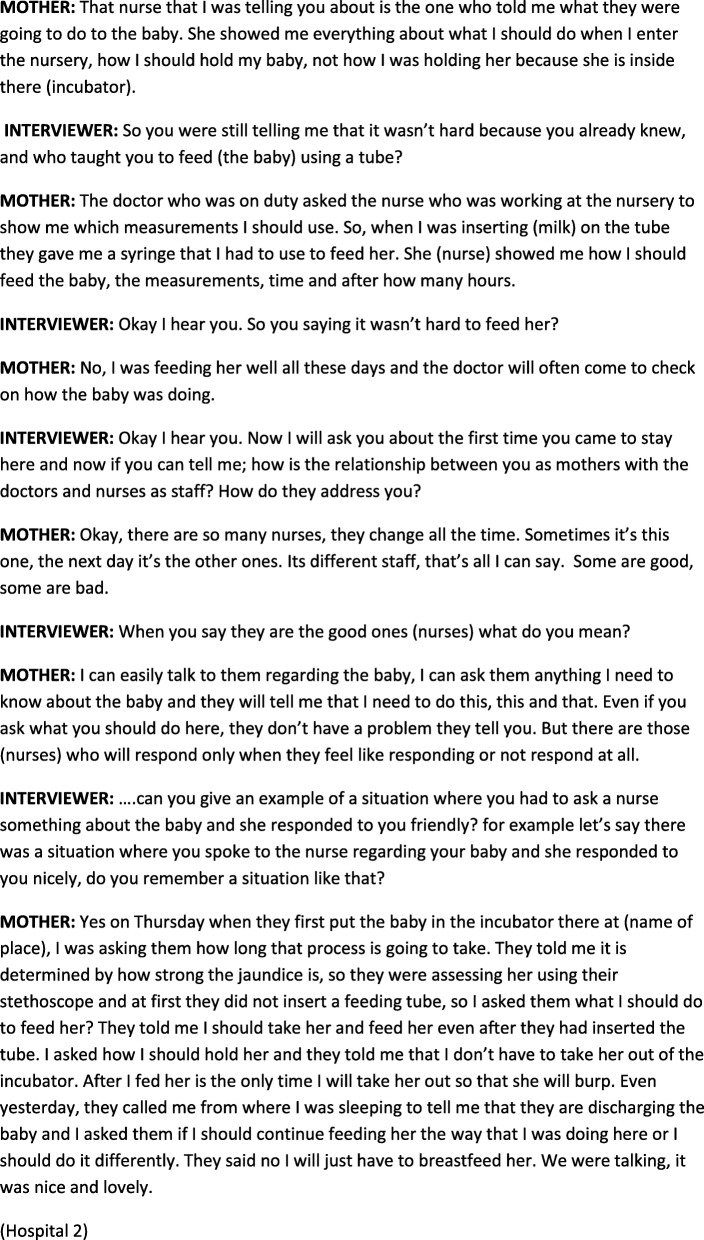
A mother’s story: communication

Mothers reported that when there was open information sharing and consultation between HWs and mothers regarding the baby’s health, they felt included in the baby’s care. Further, several mothers mentioned that when HWs were supportive in their communication, the mothers, in their turn, felt able to ask questions, raise concerns, and take a strong role in the baby’s care. Thus, effective communication from HWs led to a two-way dialogue between HWs and mothers, which helped mothers participate in the care process, and assist in the caring for the baby in a way that was an empowering and positive experience (Fig. 1).

***MOTHER:*** *So they speak nice, they explain to you that as the baby has jaundice, it is something that will end. I must make sure that I feed him and make sure that the umbilical cord is clean all the time. They are alright, they don’t have a problem because when I missed the time to wake up, they come and wake me up and I go to feed him. (Hospital 4)*

When HWs involved the mothers in the ongoing care of the baby, the mothers described feeling less anxious, more involved in the decisions and satisfied that the baby was receiving correct care. Several mothers described instances where they had informed HWs about changes in the baby’s condition or reminded the nurses about the baby’s medication.

***MOTHER:*** *When they forgot to give me medication I would go and remind them and the response that they would give me are polite and usually they will say ‘thank you for reminding us’. They will say ‘I will give you I have forgotten’, that makes it easy for you to approach them (Hospital 5).*

In contrast, some mothers reported that HWs were often rude and disrespectful to them. *‘They (nurses) sometimes yell at us, and they don’t check how the baby is’ (mother, Hospital 7).* This was confirmed from the observations where HWs were frequently seen to be disrespectful and discourteous in the way that they spoke to mothers, and to speak loudly about the baby’s condition without consideration for privacy or confidentiality. This made the mothers fearful of asking questions about their baby’s condition, and damaged the relationship between mothers and HWs, making communication difficult.

***MOTHER:*** *Because if I were to go to the sister and I tell her she forgot to give me medication for the baby and she shouts at me, I would not be confident to go to her the following day to remind her, I would be quiet (Hospital 5).*

**Fig. 2:**
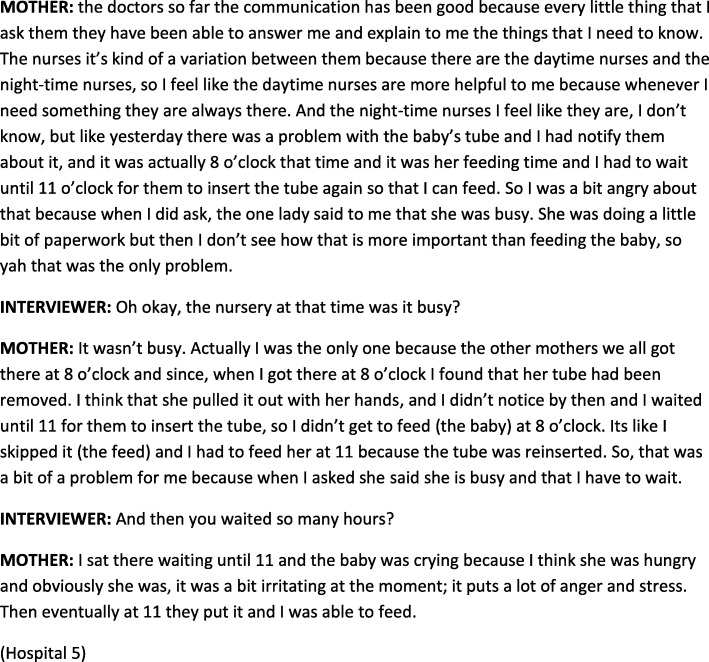
A mother’s story: neglectful care

Mothers also described that HWs did not listen to them, treat them with respect, or respond to their concerns. Observation findings also highlighted several occasions where mothers raised concerns and these were ignored or not addressed. Several mothers described occasions where HWs used their authority over mothers to make them wait for care or accept poor care. In some cases, this led to care being withheld from the baby (Fig. 2), and to serious incidents of mismanagement, as described here by a mother who returned with her baby in an ambulance from the referral hospital and was refused a bed in the neonatal unit because an administrative process had not been completed.

***MOTHER:*** *I came at night and the baby had to get some warmth; so she (nurse) said on the letter she couldn’t find the other form that should accompany it. So, she instructed me to go and ask for it on the admin department. I had put the baby on the bed but she said beds were not available at the moment. It was raining hard and there was thunder. I walked out with the baby and that did not sit well with me, it hurt me. Even today, when I think about it I get hurt. I then went to ask for that letter and they said they can keep her (the baby) because she comes from (referral hospital). I then went back to her to tell her that and she started preparing the bed for us.*

***INTERVIEWER:*** *So they made you walk with the baby in the rain?*

***MOTHER:*** *Yes, it was a thunderstorm and it was at night. (Hospital 5)*

In addition, mothers often said that, although the care of the baby was good, the HWs did not consider them as important in the care. ‘*They do not pay attention to you as a parent, they do not care if you know or you do not know’ (mother, Hospital 6).* Mothers also reported that they did not feel able to ask questions about the condition of the baby.

***MOTHER****: If you ask (questions) you become annoying (to the doctor). He just came now and took blood but he did not tell me what it is for. Maybe I must ask him again. At times they do have the care, but to a parent they do not do follow-up to make you understand what is happening. Even when a person from home is phoning to ask about the problem, how is the baby, you will say the baby is growing because the weight is increasing, but you don’t know what is going on with the baby (Hospital 6)*

##### Communication about mother’s role in caring for the baby

It is the expectation of the neonatal unit staff that mothers participate in the care of their babies, this includes expressing breastmilk, feeding their babies (either breastfeed or tube feed), changing nappies and cleaning the babies. Mothers’ activities were not always appropriate, for example in one hospital mothers were observed taking their baby’s temperatures. Mothers described feeling satisfied when HWs told them exactly what was expected of them, ‘*I like the way they are telling us how to behave, how to take care of the babies’* (mother, Hospital 4). In this way mothers were able to understand their role clearly and participate actively in the care of the baby.

***MOTHER:*** *I’m the one who puts the milk in the syringe, it’s because she’s still a bit small to breastfeed. So the nurse showed me and she told me if I have a problem…I should alert her, she will help me and I shouldn’t do anything I’m not sure about (Hospital 5).*

It was important to mothers that the reasons for the rules they were asked to follow in the neonatal unit were explained, for example hygiene practices or not placing items for their baby in the incubator. Many mothers reported that HWs did not clearly explain to them the reasons for particular actions required of them or their role in caring for their babies. As a result, mothers did not understand their role, and felt anxious and uncertain how to behave while in the neonatal unit, often relying on the support of other mothers to assist them in caring for the baby *‘I would also watch how other mothers do things, then I will do like them’ (mother, Hospital 5).* Mothers also described that HWs shouted at them for doing the wrong thing, even when they had not been given appropriate explanation or instruction.

**MOTHER***: Eish you don’t feel free [at peace], because they do not explain exactly why you should only change the baby’s nappy and not do anything...because I came to change a nappy only, they do not explain to you why you should not feed or why should not touch the baby (Hospital 6).*

##### Communication and consent for clinical procedures

Mothers expressed that they wanted to be informed about changes to clinical care and about procedures done on the baby. In particular, mothers expressed that they wanted the reasons for these to be explained and to be consulted and included in the decision-making process. In some instances, mothers described the positive experience of being asked to provide consent for procedures being done on the baby.

***MOTHER:*** *Even yesterday he (doctor) called me and told me they need to draw ‘water’ on the spine.*

***INTERVIEWER:*** *The baby’s?*

***MOTHER:*** *Yes, he drew ‘water’ from the baby’s spine and again he asked for my permission*

***INTERVIEWER:*** *Okay how does that make you feel, the fact that they ask you for permission?*

***MOTHER:*** *It makes me happy because it’s really hard if someone does something to your baby without notifying you (Hospital 5)*

In contrast, some mothers described that when they were not given information and HWs undertook procedures on the babies with no explanation, made them feel anxious, vulnerable and disempowered ‘*It is difficult when someone does something to your baby without telling you’* (mother, Hospital 7). In four hospitals it was observed that doctors carried out ward rounds when the mothers were not present, or without speaking to the mothers even when they were present. Several mothers described instances of non-consented care and this was observed in several hospitals. Overall there were many mothers who stated that, although the baby was well cared for, they felt excluded from the care of their child.

***MOTHER:*** *As I mentioned they take a very good care of the baby. The only thing that is undesirable is that they do not inform the parent. They do not inform the parent but they take good care of the children because every time when there is something that they don’t understand, they take blood and check what is the problem, they go for x-ray, ultrasound, they follow everything to the baby (Hospital 6)*

### Health worker perspectives

In contrast to the mothers perceptions of HW-mother interactions described above, HWs described that mothers frequently appeared uninterested in the care of their children, were unwilling to play their role and did not ask questions even when encouraged to do so. In particular, HWs highlighted that, not only did mothers not participate in the care of their babies, they did not follow the instructions given to them, and at times it appeared that they actively refused to do so. This often led to incorrect practices, putting the baby’s health at risk. ***‘****Yeah we do (tell the mothers) but they are not doing what we are telling them’ (Nurse, Hospital 2).* This was supported by observations where mothers were observed not washing their hands on entering the neonatal unit, and one mother who was observed washing the baby with a facecloth brought in from outside the unit, despite being repeatedly instructed not to do so. HWs expressed frustration saying that mothers appear to do the opposite of what they are asked to do to protect the health of their babies, thereby risking the health of all babies on the unit. As a result, in some cases staff on the neonatal unit took over the care of the baby, removing the mother’s responsibilities in providing care for her baby.

***INTERVIEWER:*** *how do you as doctors cope with that (mothers not complying with instructions)..?*

***DOCTOR:*** *Well, unfortunately we can’t play PI (private investigator) all the time, you know, but what we try to do is to make sure that the baby that is in the nursery is taken care of, as possible as we can. If the mother is not playing her part, we take the responsibilities away from her and we try and take care of the baby (Hospital 5)*

HWs expected mothers to take an active role by asking about the progress of the baby. In particular, nurses expected mothers to ask about their baby’s health from the doctor to demonstrate their interest in the health of the baby but mothers appeared unaware of this expectation. During observations it was noted that nurses became annoyed when mothers only asked about the baby’s health after the doctor was gone, and at times nurses ignored the mother’s questions and told her she should ask the doctor when he/she next came around. However, when asked directly, HWs were able to explain this behaviour in terms of the African culture, where asking questions may indicate a lack of respect. ‘*with the African culture I think it’s that respect, part of respect for them (mothers) is to not question anything, not to ask anything, just to accept whatever is being done’ (doctor, Hospital 6)*

***DOCTOR:*** *It’s still a situation where you actually have to call the mother, explain things to her because they would not ask things especially to us doctors. Sometimes…sisters will overhear them (mothers) talking …then they will start complaining about what they don’t know and all their fears, so they won’t talk to you, they will talk to each other (Hospital 6).*

In addition, in several hospitals HWs described mothers giving incorrect and misleading information about the health and progress of their babies. This was interpreted by HWs as the mothers ‘lying’ about the condition of the baby in hope of being discharged sooner from the hospital.

***Nurse:*** *I think the problem is they want to go home. I think if they express their feelings, their problems, that will cause them to stay in the hospital for long time do every time, (so) they just say no, no problem (Hospital 2).*

In one case, a doctor described how mothers smuggled in formula milk to the neonatal unit to feed their babies, and pretended that it was expressed breastmilk.

***DOCTOR****: it’s because when they walk in, you see all of them walking in and you aah “but you went from no milk to now so much milk and it’s so white”. And we actually found out that, no, they (are) actually sneaking in formula (milk), so now we have asked them to express when they’re here so that we can actually see them, so now they have to express in the ward that’s why we started with that rule (Hospital 5)*

## Discussion

This study used a strong study design with observation and interview data collected over an extended period of five days and triangulated to validate the findings. The findings highlight the importance of information sharing between HWs and mothers of babies admitted in the neonatal unit. We contrast the positive communication reported by many mothers which led to them feeling empowered and participating actively in the care of their babies, with incidents of poor communication leading to misunderstandings and poor care. Where care was provided in partnership with mothers, mothers were able to make a positive contribution, feeding, changing nappies and observing the condition of their babies, and even reminding overworked nurses when medication was due. In contrast, poor communication, rudeness and disrespectful behaviour of HWs was frequently described by mothers, leading to their feeling anxious and excluded from the baby’s care, unsure how to behave and unwilling to ask questions. In extreme cases mothers reported that nurses appeared to purposely delay or withhold care from babies leading to incidents of severe mismanagement. In contrast, health workers described mothers failing to take part in their babies care, ignoring instructions, and even misleading health workers about their babies’ progress in order to be discharged earlier. Our findings suggest that interpersonal aspects of care are not only important for patient satisfaction, but may also have serious implications for the quality of care provided in the neonatal unit, and the potential to impact the outcomes for these babies.

Communication is considered effective when the receiver of the message understands the message in the way that was intended by the sender [35]. Our results suggest that ineffective communication between mothers’ and HWs led to their respective actions being perceived in different and often negative ways. For example, when HWs observed that mothers were reluctant to ask questions from the doctor, this was interpreted as mothers being uninterested in their baby’s health. However, there were clearly other possible reasons, including that mothers were disempowered or fearful, or wanted to show respect to the doctor, and health workers themselves were able to provide alternative explanations when directly asked. Ineffective communication led to misunderstandings that further undermined communication and reinforced undesirable behaviour, and participants described instances where poor communication led directly to poor quality of care. Thus, poor communication powerfully undermines provision of quality care in the neonatal unit. Open and respectful communication between mothers and HWs could resolve such misunderstandings, and could form the basis of shared decision making [36, 37]. Further, our findings suggest that when such dialogue occurs mothers can be empowered to participate in caring for the baby, thereby improving quality of care, patient satisfaction and even reducing HWs workload. A previous study in South African neonatal units also showed inadequate communication, with HWs often making clinical decisions without consulting parents, despite parents wanting to be involved in decision-making [36]. HWs, particularly doctors, are often dominant in decision making [19, 38], and may not fully believe in mothers’ ability to make informed decisions [19]. Therefore, HWs should critically evaluate their commitment to patient participation in the life-changing decisions made in the neonatal unit [37]. Communication skills have also been shown to be lacking among HWs [16], and there is a need to develop inter-personal and communication skills among HWs if there is to be a shift towards more respectful care. Communication and counselling skills can be improved by training [39], and this should be a priority for HWs providing care for newborn babies.

In recent years there has been increasing focus on incidents of disrespectful behaviour towards pregnant women and mothers, including scolding, insulting remarks and withholding of care, which have been described in several African settings [16]. The important contribution that patient experiences make to quality of care, and the importance of negative experiences as a barrier to seeking care must be addressed if coverage of facility-based maternity care is to be increased [16]. This has led to the development of a respectful maternity care charter setting out the rights of childbearing women [21], and since the mother and baby dyad are inseparable, it has been proposed that the right to respectful care should be extended to care of the baby [27]. Our findings suggest that disrespectful care, lack of confidentiality, non-consented care and neglectful care also occur in neonatal units in our setting, and supports the suggestion that the charter be extended to include neonatal care. It is not clear whether our findings can be generalised beyond KZN but similar findings from other studies and the relatively large numbers of participants and observations suggests that these findings are unlikely to be isolated.

In several instances HWs described mothers’ behaviour as being uncaring, with mother’s reportedly giving false information and smuggling in formula milk in order to be discharged earlier. Other studies confirm poor concordance between what doctors say and what parents hear and understand [38], and high levels of maternal anxiety can be associated with poor recall of counselling messages [40]. This should be seen in the context where admission of a baby to the neonatal unit leads to a sudden, often unexpected, separation of the mother from her baby, and may be associated with feelings of despair, hopelessness and disappointment leading to prolonged maternal distress [41]. Admission to the neonatal unit may disrupt bonding between mother and baby, mothers may lose the sense of their maternal role and even be fearful of developing bonds with a baby that may die [42, 43]. In our setting many mothers lack resources to visit home or receive visits from their family and, since they are breastfeeding, may be isolated at the hospital for long periods with little or no emotional support. Mothers require help to adapt and develop their parental role and bond with the baby. Several studies suggest that support provided by HWs plays a critical role in coping with this distress and anxiety [41, 43]. In particular, the first visit to the neonatal unit is important with many mothers perceiving the environment as threatening, and can be overwhelming, even leading to difficulties for the mother in recognizing the baby as her own [42, 43]. In contrast, involvement in the care of the baby promotes the mothers self-esteem, empowers her within that environment, allays anxiety and makes her more secure in her identity as a mother and encourages bonding [41]. HWs can provide information support and guidance, and actively promote confidence in the interaction with the newborn baby [42]. Building the mothers’ confidence and involving her in the baby’s care can have long-term benefits for the well-being of mother and baby, so it is important that HWs actively promote mother-baby bonding and see this as a key role. In our study many mothers described not being informed of the routines in the nursery or about their role, leading to unnecessary stress and anxiety. Further, behaviours that HWs describe as uncaring could be related to lack of bonding and the mother’s difficulties in coming to terms with the change in her situation. In our setting mothers frequently rely on HWs and other mothers for support, but HWs in our study expressed little awareness of the importance of involving the mother to promote bonding, and the importance of bonding to achieving positive long term outcomes for the baby. Development of strong bonds between mother and baby is particularly important when many mothers are young and inexperienced, and living in low income households, as is often the case in our setting. Building HW skills and awareness to support mothers to bond with their babies is crucial, and should be specifically included in training of HWs providing newborn care.

### Study limitations

Limitations of the study included exclusion of very small hospitals for logistical reasons, these hospitals may face particular challenges that were not represented in this study. We also did not conduct observations during the evening or during the night or observe HWs on duty after hours. Fathers were not included as participants, this was as a result of cultural practices that frequently exclude fathers from contact with the babies in the first weeks of life, but including those fathers involved in the baby’s care may have added an additional perspective.

The presence of the observer may have changed the behaviour of participants on the neonatal unit but the prolonged period of observation mitigated against this. Although interviews with mothers were conducted privately it was possible that mothers were reluctant to criticise the care received while their babies were still admitted in the unit. Similarly, HWs were interviewed at their place of work and may have felt unable to speak freely about colleagues or hospital managers.

## Conclusion

Good communication and dialogue between mothers and HWs caring for newborn babies has a critical role to play in building mothers’ confidence, promoting bonding and participation of mothers in the care of their baby and may have long term benefits for the health and well-being of both the mother and her baby. HWs have a strong role to play in supporting and caring for mothers but may lack awareness and skills to provide this role. These findings will provide a strong motivation to the DoH to support HWs training to improve communication and increased awareness of the importance of the HWs role in providing care to both members of the mother-baby dyad.

## Data Availability

The data is available from the main author on reasonable request.

## Abbreviations

CEO: Chief Executive Officer
CPAP: Continuous Positive Airways Pressure
DHIS: District Health Information System
DoH: Department of Health
HW: Health worker
KINC: KwaZulu-Natal Initiative for Newborn Care
KZN: KwaZulu-Natal
NN: Neonatal
QoC: Quality of Care
WHO: World Health Organization
